# HuR-Dependent Editing of a New Mineralocorticoid Receptor Splice Variant Reveals an Osmoregulatory Loop for Sodium Homeostasis

**DOI:** 10.1038/s41598-017-04838-8

**Published:** 2017-07-06

**Authors:** Ingrid Lema, Larbi Amazit, Khadija Lamribet, Jérôme Fagart, Anne Blanchard, Marc Lombès, Nadia Cherradi, Say Viengchareun

**Affiliations:** 1 Inserm U1185, Fac Med Paris Sud, Université Paris-Saclay, F-94276 Le Kremlin-Bicêtre, France; 2 UMS 32, Le Kremlin-Bicêtre, 94276 France; 3 Inserm, Centre d’Investigations Cliniques 9201, F-75015 Paris, France; 4 Assistance Publique-Hôpitaux de Paris, Hôpital de Bicêtre, Service d’Endocrinologie et des Maladies de la Reproduction, Le Kremlin Bicêtre, F-94275 France; 5 Institut National de la Santé et de la Recherche Médicale, Inserm U1036, Grenoble, F-38000 France; 6 Commissariat à l’Energie Atomique et aux Energies Alternatives, Institut de Biosciences et Biotechnologies de Grenoble, Laboratoire Biologie du Cancer et de l’Infection, Grenoble, F- 38000 France; 7 Université Grenoble Alpes, Unité Mixte de Recherche-S1036, Grenoble, F-38000 France

## Abstract

Aldosterone and the Mineralocorticoid Receptor (MR) control hydroelectrolytic homeostasis and alterations of mineralocorticoid signaling pathway are involved in the pathogenesis of numerous human diseases, justifying the need to decipher molecular events controlling MR expression level. Here, we show in renal cells that the RNA-Binding Protein, Human antigen R (HuR), plays a central role in the editing of MR transcript as revealed by a RNA interference strategy. We identify a novel Δ6 MR splice variant, which lacks the entire exon 6, following a HuR-dependent exon skipping event. Using isoform-specific TaqMan probes, we show that Δ6 MR variant is expressed in all MR-expressing tissues and cells and demonstrate that extracelullar tonicity regulates its renal expression. More importantly, this splice variant exerts dominant-negative effects on transcriptional activity of the full-length MR protein. Collectively, our data highlight a crucial role of HuR as a master posttranscriptional regulator of MR expression in response to osmotic stress. We demonstrate that hypotonicity, not only enhances MR mRNA stability, but also decreases expression of the Δ6 MR variant, thus potentiating renal MR signaling. These findings provide compelling evidence for an autoregulatory feedback loop for the control of sodium homeostasis through posttranscriptional events, likely relevant in renal pathophysiological situations.

## Introduction

The Mineralocorticoid Receptor (MR) belongs to the nuclear receptor superfamily. This ligand-activated transcription factor mediates aldosterone action in the distal nephron, where it participates to tight regulation of Na^+^ reabsorption by stimulating expression of ionic transporters^1^. Pivotal role of MR was demonstrated in mice carrying homozygous *Nr3c2* null mutation that died 10 days after birth presenting with a profound salt wasting phenotype^2^. In humans, MR mutations are responsible for type I pseudohypoaldosteronism (PHA1), a rare disease causing an important neonatal salt wasting^3^. Conversely, overactivation of MR signaling pathway leads to numerous deleterious effects in humans, such as Na^+^ retention, hypervolemia, high blood pressure that are responsible for kidney and heart damage^4, 5^. Thus, compelling studies underscored the importance of controlling MR expression level and activity and of deciphering molecular mechanisms regulating notably renal MR expression. Along this line, numerous studies emphasized the major impact of posttranscriptional mechanisms in the control of mRNA turnover, particularly in chronic kidney diseases, as recently reviewed by Feigerlová^6^. In this context, our group reported that renal MR expression is tightly regulated by variations of extracellular tonicity through posttranscriptional mechanisms. Indeed, we recently showed that hypertonic stress strongly induces expression of the RNA-Binding Protein (RBP) Tis11b (tetradecanoyl phorbol acetate inducible sequence 11b), leading to MR transcript degradation, blunted responses to aldosterone stimulation and impaired MR signaling^7^. On the opposite, hypotonic stress triggers rapid nuclear export of the RBP Human Antigen R (HuR) to the cytoplasm, where HuR interacts with MR 3′-untranslated region (3′-UTR) to stabilize and increase MR transcript and protein levels, thereby enhancing MR signaling (see Lema *et al*., 2017, submitted). HuR is an ubiquitous RBP that belongs to the Hu protein family and is recognized as the major stabilizing RBP of short-lived RNA stability and expression^8–10^. Importantly, HuR is also considered to be an important regulator of alternative splicing, either directly, by promoting exon skipping, or indirectly, by modulating the expression or the action of splicing factors such as TIA-1/TIAR^11–13^. Alternative splicing is now described as a general process impacting more than 90% of human genes based on deep sequencing studies^14, 15^, thus minimizing the general dogma of paucity of such molecular events in humans. Alternative splicing now represents the prominent mechanism in generating diversity in gene products for higher eukaryotes by allowing differential inclusion or exclusion of exons and introns in the mature RNA, thus leading to the expression of various transcripts and proteins resulting from an unique gene sequence. RBP are important class of alternative splicing regulators that play pivotal roles in posttranscriptional gene regulation^16^, capable of binding to pre-mRNA on specific *cis*-acting sequences located either in introns named ISS (intronic sequence silencers) or ISE (intronic sequence enhancers) or in exons named ESE (exonic sequence enhancers) or ESS (exonic sequence silencers). Binding of RBP to these specific sequences modulates spliceosome action on pre-mRNA^17, 18^. Therefore, deregulation of alternative splicing appears to be a determinant factor in various pathological disorders like cancer and neurodegenerative diseases^19, 20^.

With respect to MR, several splice variants have been reported in both human and rat species^21–24^. These variants were detected with different PCR strategies (screening of human cDNA libraries or use of degenerate primers) in numerous MR target tissues^21, 22^ but their expression level was relatively low compared to that of the full-length (FL) MR transcript. Along this line, we previously identified the human Δ5.6 MR^21^ which exerts a dominant-positive effect on the FL MR. However, the molecular mechanisms underlying such alternative splicing (insertion, deletion or exon-skipping) were not described in these previous studies. Moreover, the biological significance of such splice variants has not been clearly examined.

In the present study, we now identify, by an exon-specific PCR strategy, a novel mouse Δ6 MR splice variant, lacking exon 6, resulting from a HuR-dependent alternative splicing event. This MR transcript isoform is a constitutive transactivator insensitive to aldosterone action, which exerts dominant-negative effects on the activity of FL MR protein. We also demonstrate that hypotonicity decreases HuR nuclear localization thereby reducing the relative expression level of Δ6 MR while increasing FL MR expression. This HuR-dependent editing of MR transcript converges to promote renal aldosterone responsiveness and, presumably, Na^+^ reabsorption in renal cells. Collectively, our previous work reveals a novel regulatory mechanism by which transient HuR nucleocytoplasmic shuttling facilitates rapid adaptive responses to hypotonic stress in renal cells (see Lema *et al*., 2017, submitted). This two-level posttranscriptional regulation constitutes the first demonstration linking processing and stabilization of a nuclear receptor mRNA with major potential pathophysiological consequences, particularly in a context of aldosterone-related kidney diseases, hypertension or mineralocorticoid resistance.

## Results

### Identification of a new Δ6 MR splice variant

**Figure 1:**
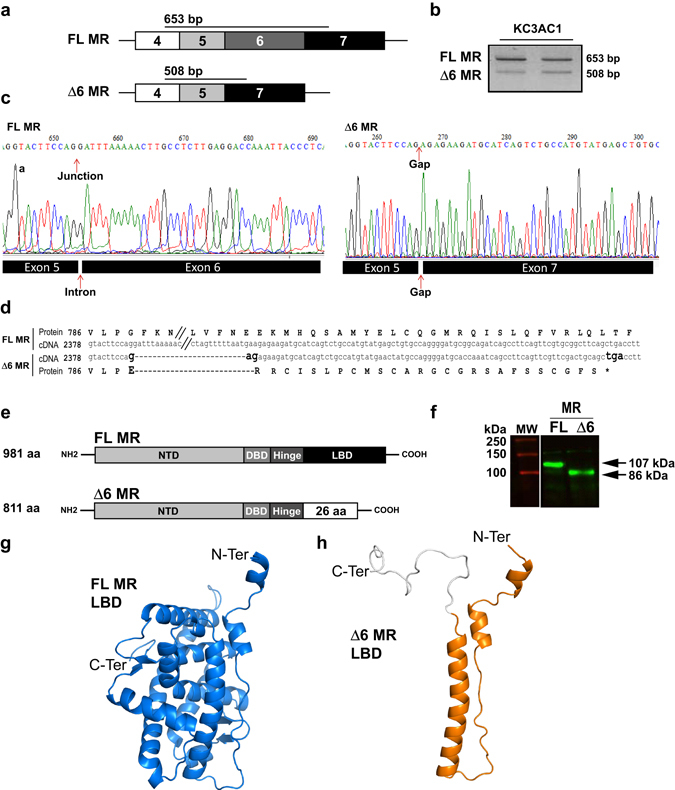
An exon skipping event generates a new MR splice variant. (**a**) Schematic representation of exons 4 to 7 of *Nr3c2* gene encoding for mouse MR. Δ6 MR lacks exon 6 (**b**) RT-PCR amplification of FL MR (653 bp) and Δ6 MR (508 bp) from two KC3AC1 cDNA samples. (**c**) Alignment of the FL MR and Δ6 MR sequences between exons 5 and 7. Nucleotides 2388 to 2532 (exon 6), have been omitted from the FL MR sequence (// symbol). Exon 6 deletion introduces a frameshift and creates a premature stop codon, leading to a truncated MR protein. (**d**) Sequencing of the FL and Δ6 MR transcripts. Murine FL MR and Δ6 MR were amplified by RT-PCR from differentiated KC3AC1 cells and inserted into the pGEMT-easy vector. Chromatograms of FL MR (left panel) and Δ6 MR (right panel) revealed that the Δ6 MR splice variant lacks the entire exon 6. (**e**) Structure of FL MR and Δ6 MR proteins: the premature stop codon leads to deletion of most of the LBD domain in the Δ6 MR. (**f**) *In vitro* translation of plasmids encoding FL MR and Δ6 MR, followed by western blotting with 39 N MR antibody. FL MR (107 kDa) and Δ6 MR (86 kDa) proteins are visualized. (**g**,**h**) Three-dimensional models illustrating structures of FL MR (**g**) and Δ6 MR LBD (**h**). Helices and β strands are shown, (PyMOL Molecular Graphics System).

Given that alternative splicing has been previously shown to generate MR splice variants in humans and rats, we searched for new MR transcript isoforms using PCR amplification with exon-specific oligonucleotides of reverse-transcribed cDNA isolated from murine renal KC3AC1 cells (Fig. 1a). Two amplicons of 653 bp and 508 bp were generated, potentially corresponding to the FL MR and to an unknown splice variant of MR, respectively (Fig. 1b). Further sequencing of these PCR fragments identified a MR splice variant lacking the entire exon 6 of MR transcript, referred to as Δ6 MR (Fig. 1c and d). The skipping of exon 6 leads to an open reading frameshift, generating a premature stop codon. Sequence comparison revealed that the Ligand-Binding Domain (LBD) of murine Δ6 MR is conserved up to the Pro788 residue (Pro794 in human MR, hMR), with the rest of the LBD (residues 789 to 978 in the murine FL MR) replaced by a shorter (26 amino acids) C-terminal tail, resulting in a truncated protein (86 kDa for Δ6 MR and 107 kDa for FL MR; Fig. 1e), as shown by *in vitro* translation of the Δ6 MR transcript followed by western blotting (Fig. 1f). We generated three-dimensional homology models of the murine FL MR and Δ6 MR, using the crystal structure of the hMR LBD as a template^25^. As shown in Fig. 1g, FL MR LBD adopts the canonical fold of the nuclear receptor LBD, encompassing the Nter-H1, H1, and H3 to H12 helices. In sharp contrast, Δ6 MR LBD contains only the Nter-H1, H1 and H3 helices (Fig. 1h). The short C-terminal tail, which has no structural homolog, is modelled as an unstructured coil. The H1 and H3 helices are part of the LBD scaffold and the absence of the rest of the domain probably results in an unstable truncated LBD.

### The ligand-independent Δ6 MR is a dominant-negative effector of FL MR

**Figure 2:**
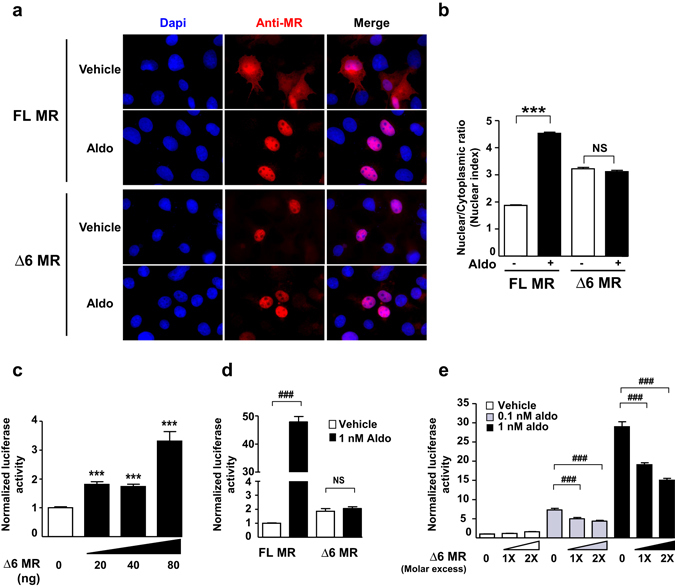
Δ6 MR is a ligand-independent transactivator and exerts dominant-negatif effects on FL MR. (**a**) Analysis of subcellular trafficking of the Δ6 MR splice variant. For immunocytochemistry, COS7 cells were cultivated in 4-well chamber slides (Sarstedt). Cells were transfected with 500 ng of FL MR- or Δ6 MR-encoding vector in the presence of Lipofectamine 2000 and incubated for 1 hour with ethanol (vehicle) or 10 nM aldosterone (Aldo). Cells were fixed and processed for MR immunodetection with the 39 N anti-MR antibody. Representative images of the subcellular localization of MR FL and MR Δ6 before and after hormone treatment. After aldosterone stimulation, FL MR translocated to the nucleus, whereas Δ6 MR was already present in the nucleus, even in the absence of the ligand. The nuclei were stained with DAPI (blue). (**b**) HuR immunodetection was coupled with an automated high-throughput microscopy (HTM) analysis, to quantify the subcellular trafficking of this protein. The results are expressed as the mean ratio of nuclear to cytoplasmic fluorescence ± SEM (*n* > 800 cells). Bar, 25 µm. ***P < 0.001; NS = not significant (Mann-Whitney U-tests). (**c**,**d**,**e**) HEK 293 T cells were transfected as described in the Materials and Methods. (**c**) HEK 293 T cells were transfected with various amounts of the plasmid encoding Δ6 MR (0 to 80 ng). The following day, firefly-luciferase activities were measured and normalized relative to β-galactosidase activities. The data shown are means ± SEM of two independent experiments (*n* = 24). ***P < 0.001 (Mann-Whitney U-tests). (**d**) After transfection, HEK 293 T cells were incubated for 24 hours with ethanol (vehicle) or with 1 nM aldosterone. Firefly-luciferase activities, normalized to β-galactosidase activities, are means ± SEM (*n* = 8). ***P < 0.001; ^*###*^ *P* < 0.001 (Mann-Whitney U-tests). (**e**) Δ6 MR acts as a dominant-negative transactivator of FL MR. HEK 293 T cells were transfected with FL MR alone or with a one-fold (20 ng) or two-fold excess (40 ng) of Δ6 MR. Cells were incubated for 24 hours with ethanol (vehicle) or with 0.1 or 1 nM aldosterone. Luciferase activities were measured as described above. Data are means ± SEM of three independent experiments (*n* = 24). ***P < 0.001; ^*###*^ *P* < 0.001 (Mann-Whitney U-tests).

To characterize this new MR splice variant, we analyzed subcellular distribution of FL MR and Δ6 MR in response to aldosterone by transfecting COS7 cells with their respective vector constructs. Immunocytochemistry showed that aldosterone induces a clear relocalization of FL MR to the nucleus (Fig. 2a). Quantification of the nuclear/cytoplasmic ratio (N/C ratio) using automated high-throughput microscopy (HTM)^26^ revealed that aldosterone treatment for 1 hour leads to a robust increase in N/C ratio, consistent with the import of FL MR to the nucleus, as previously described (Fig. 2b)^27, 28^. Interestingly, immunocytochemistry and HTM quantitification showed that Δ6 MR is mostly present in the nucleus, both in the absence and presence of aldosterone (Fig. 2a and b). We then investigated the functional properties of the Δ6 MR splice variant. As shown in Fig. 2c, in absence of hormone stimulation, Δ6 MR was capable of activating transcription of the glucocorticoid response element 2 (GRE2)-driven luciferase gene. Interestingly, basal transactivating activity of Δ6 MR was twice as strong as that of FL MR (***P < 0.0002, Fig. 2d). In contrast, aldosterone increased FL MR-mediated GRE2-driven firefly-luciferase activity by 50-fold, whereas the Δ6 MR variant was insensitive to aldosterone stimulation (Fig. 2d), consistent with a ligand-independent transactivating function of this LBD-lacking receptor. We also investigated whether the presence of the Δ6 MR variant could affect FL MR activity. HEK293T cells were transfected with various amounts of Δ6 MR-encoding plasmid and a constant amount of FL MR-encoding plasmid. The use of equimolar concentrations of FL MR- and Δ6 MR-encoding plasmids resulted in significantly lower levels of aldosterone-stimulated luciferase activity (40% lower than those in the absence of the Δ6 MR-encoding plasmid; Fig. 2e). This effect was dose-dependent, as demonstrated by reduction of FL MR activity in the presence of a two-fold excess Δ6 MR-encoding plasmid independently of aldosterone concentration (Fig. 2e).

### Δ6 MR expression and its regulation by extracellular tonicity

**Figure 3:**
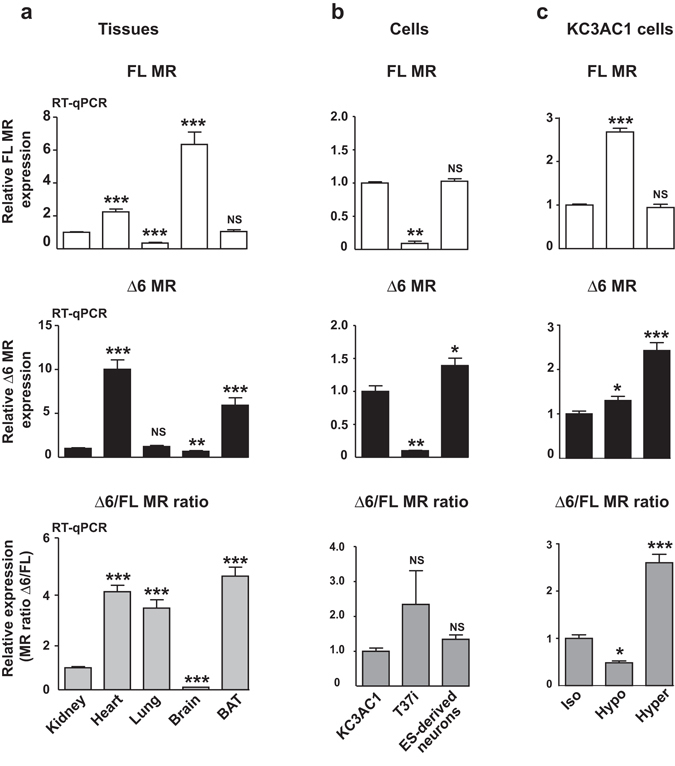
Δ6 MR is expressed in mouse MR-expressing tissues and cells, and is regulated by extracellular tonicity. (**a**,**b**) RT-qPCR analysis of MR expression (FL and Δ6 MR). The FL MR and Δ6 MR transcripts were reverse-transcribed and amplified with Taqman probes from several mouse epithelial and non-epithelial MR-expressing tissues (kidney, heart, lung, brain and brown adipose tissue, BAT) or cell lines (KC3AC1, T37i cells, ES-derived neurons). The expression of FL MR or Δ6 MR, normalized relative to *18s* rRNA, is expressed as a fold-induction relative to the level of expression in the kidney or in KC3AC1 cells, arbitrary set at 1. The Δ6/FL MR ratio was also determined for each tissue or cell line. The data shown are means ± SEM (*n* = 12–18) of two and three independent experiments, for each tissue and cell line, respectively. **P < 0.01; ***P < 0.001; NS = not significant (Mann-Whitney U-tests). (**c**) MR expression (FL MR and Δ6 MR) was also analyzed by RT-qPCR in KC3AC1 cells subjected to isotonic (Iso), hypotonic (Hypo) or hypertonic (Hyper) conditions for 6 hours. FL MR or Δ6 MR expression is presented as described above (fold-induction relative to the level of expression in control cells [Iso], arbitrarily set at 1). The Δ6/FL MR ratio was also determined for each set of conditions. The data shown are means ± SEM (*n* = 16) of two independent experiments. *P < 0.05; **P < 0.01; ***P < 0.001; NS = not significant (Mann-Whitney U-tests).

Expression of Δ6 MR variant was further analyzed in MR-expressing tissues and cells, by RT-qPCR with exon-specific primers and Taqman probes for the selective quantification of each MR transcript isoform. As expected, FL MR transcript was detected in the kidney, lung, and brown adipose tissue (BAT) (Fig. 3a, upper panel). Relative expression levels of Δ6 MR transcript were low compared to the FL MR. Nevertheless, a differential pattern of Δ6 MR expression was observed in these MR-expressing tissues (Fig. 3a, middle and lower panels), with the highest expression levels observed in the heart and BAT, suggesting the conservation of these alternative splicing events for the regulation of MR expression. We also measured Δ6 MR expression in KC3AC1 renal cells, T37i brown adipocytes and embryonic stem cell-derived neurons (Fig. 3b). As expected, FL MR transcript levels were highest in renal and ES-derived neuronal cells. Δ6 MR expression levels in brown adipocytes were about one tenth of those in renal cells. We also investigated the possible effect of osmotic stress on this splicing process, by exposing KC3AC1 cells to isotonic, hypotonic (150 mOsmol/l) and hypertonic (500 mOsmol/l) media. FL MR expression level was drastically increased by hypotonic stress, whereas Δ6 MR variant expression was only slightly increased, resulting in a low Δ6/FL MR ratio (Fig. 3c). Conversely, hypertonicity increased expression level of Δ6 MR, resulting in a high Δ6/FL MR ratio. These data indicate that hypoosmotic stress modulates renal MR transcript splicing and expression to promote aldosterone responsiveness through fully functional FL MR signaling.

### Alteration of the osmotic gradient modulates renal Δ6 MR expression *in vivo*

**Figure 4:**
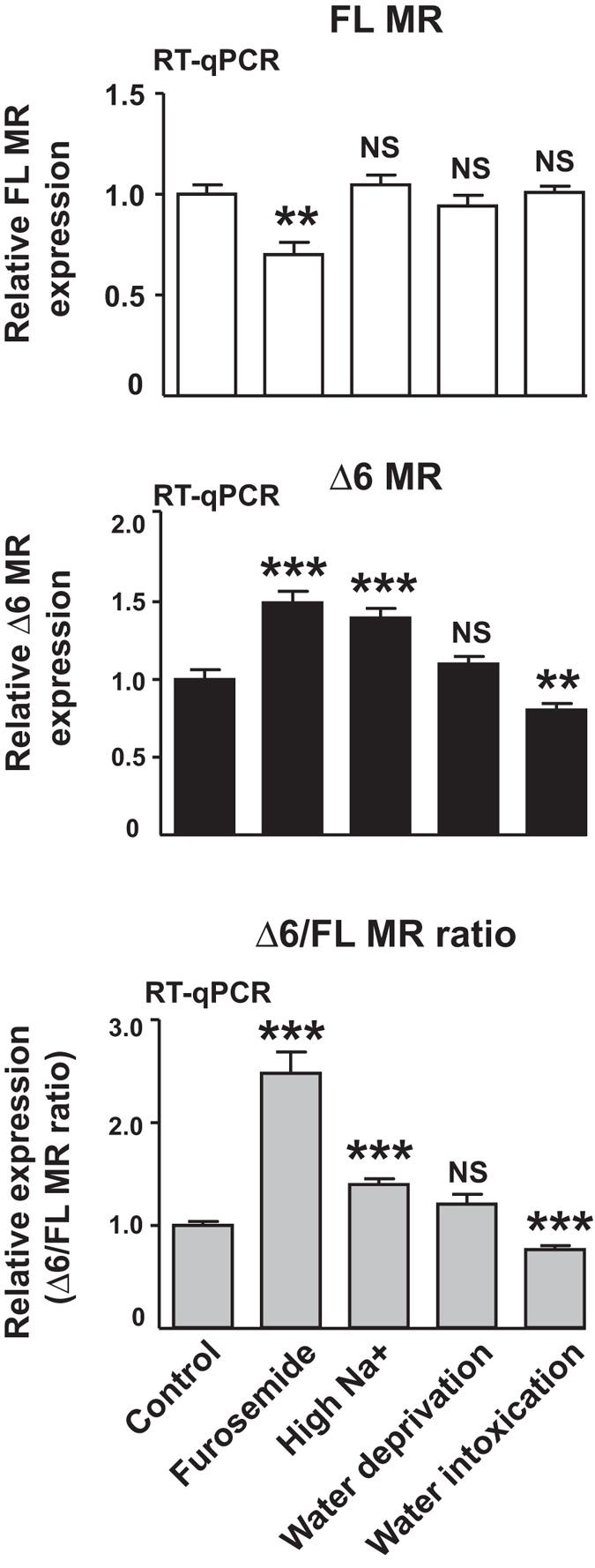
Alteration of osmotic gradient modulates renal Δ6 MR expression *in vivo*. RT-qPCR analysis of MR expression (FL MR and Δ6 MR) in the kidneys of mice subjected to furosemide treatment, a high Na^+^ diet, water deprivation or water intoxication. The FL MR and Δ6 MR transcripts were reverse-transcribed, amplified specifically with appropriate primers and quantified with Taqman probes. Levels of mRNA for the FL MR or Δ6 MR were normalized relative to *18s* rRNA and expressed as a fold-induction relative to the level of expression in the kidneys of control animals, arbitrarily set at 1. The data shown are means ± SEM (*n* = 10–24) of two independent experiments. The Δ6/FL MR ratio was also determined for each set of conditions. **P < 0.01; ***P < 0.001; NS = not significant (Mann-Whitney U-tests).

We evaluated the pathophysiological relevance of our findings, by analyzing renal Δ6 MR expression in mice subjected to various experimental challenges, such as furosemide exposure, a high-sodium diet, water deprivation or water intoxication. These conditions are known to modify sodium delivery in the distal parts of the nephron. Furosemide, a loop diuretic, inhibits NKCC2 and increases acute sodium delivery to the cortical collecting ducts and luminal osmolarity. As shown in Fig. 4, furosemide significantly decreased renal FL MR levels and increased Δ6 MR splice variant levels, resulting in a higher Δ6/FL ratio. In sharp contrast, neither high-sodium diet nor overnight water deprivation affected renal FL MR levels, whereas Δ6 MR levels were increased by a high-sodium diet and remained unchanged following overnight water deprivation. Therefore, we observed a significant increase in Δ6/FL ratio in kidneys of animals fed a high-sodium diet, whereas this ratio was not modified by water deprivation (Fig. 4, lower panel). Water intoxication, a condition of relative hypotonicity of luminal cortical fluid, did not modify total renal MR levels, but it significantly decreased Δ6 MR levels, resulting in a decrease in Δ6/FL ratio consistent with our observations *in vitro* for KC3AC1 cells exposed to hypotonic stress (see Fig. 3c, lower panel). Taken together, these results indicate that, *in vivo*, changes in hydroelectrolyte balance radically alter renal MR expression in terms of mRNA levels for both FL MR and its splice variant Δ6 MR.

### HuR is instrumental in hypotonicity-induced Δ6 MR editing

**Figure 5:**
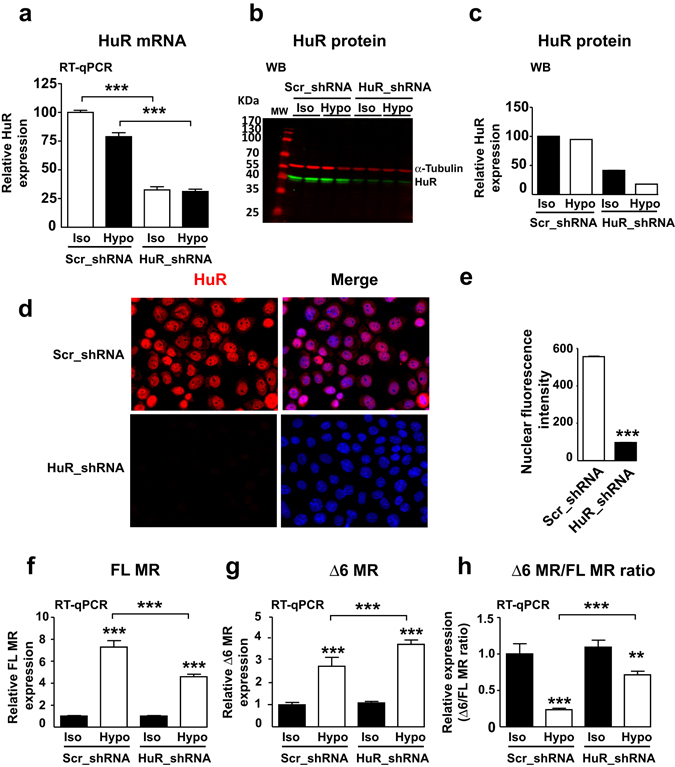
HuR favors exon 6 inclusion in MR transcript, thereby promoting the production of the FL MR under hypotonic conditions. KC3AC1 cells stably expressing scrambled (Scr) or HuR short hairpin RNA (shRNA, clone 14), were cultured under isotonic (Iso) or hypotonic (Hypo) conditions for 6 hours. (**a**) HuR mRNA levels were determined by RT-qPCR and normalized relative to *Gapdh* mRNA levels. The values shown are means ± SEM (*n* = 20–24). ***P < 0.001 (Mann-Whitney U-tests). (**b**, **c**) HuR protein levels were analyzed by WB (**b**) in KC3AC1 cells expressing Scr_ or HuR_shRNA. α-tubulin was used as a loading control. Relative levels of HuR, based on the quantification of band intensity, are shown in (**c**). Data shown are means of two independent determinations. (**d**) Immunodetection of HuR in KC3AC1 cells stably expressing Src_shRNA or HuR_shRNA. The nuclei were stained with DAPI. (**e**) HuR expression was quantified by automated high-throughput microscopy, as described in the Materials and Methods section. The results are expressed as the mean nuclear fluorescence intensity. HuR expression was decreased by ~90% in cells transfected with the HuR_shRNA. ***P < 0.001, *n* > 5000 cells in each set of conditions (Mann-Whitney U-tests). (**f**,**g**) RT-qPCR analysis of FL MR (**f**) and (**g**) Δ6 MR in KC3AC1 cells stably expressing either Scr_ shRNA or HuR_shRNA, cultured under isotonic (Iso) or hypotonic (Hypo) conditions for 6 hours. The FL MR and Δ6 MR transcripts were reverse-transcribed, amplified and quantified in a specific manner, as described above. Their levels were normalized relative to *Gapdh* mRNA. (**h**) The Δ6 MR/FL MR ratio is also presented. Note that knocking down HuR expression with HuR_shRNA significantly limited the hypotonicity-induced decrease in Δ6 MR/FL MR ratio. The data shown are means ± SEM (*n* = 26 to 36) of three independent experiments. **P < 0.01; ***P < 0.001 (Mann-Whitney U-tests).

To unambiguously demonstrate the direct relationship between expression of HuR and Δ6 MR splice variant, we generated KC3AC1 cell lines stably transfected with short hairpin RNA (shRNA) to knockdown HuR expression. KC3AC1 cells expressing scrambled (Scr_shRNA) or HuR (HuR_shRNA) shRNA were incubated under isotonic or hypotonic media. Silencing efficiency was determined by RT-qPCR and western blotting. As showed in Fig. 5, hypotonic stress did not affect HuR mRNA or protein levels. HuR mRNA levels were significantly decreased by HuR_shRNA under isotonic or hypotonic conditions, resulting in a parallel decrease in the level of the protein (Fig. 5a–c). This decrease in HuR levels was confirmed by immunocytochemistry coupled to HTM quantification (Fig. 5d–e). RT-qPCR analysis revealed that hypotonicity increases FL MR and Δ6 MR levels in KC3AC1 cells stably transfected with Scr_shRNA or HuR_shRNA. However, the knockdown of HuR expression significantly decreased FL MR levels whilst increasing those of Δ6 MR Fig. 5f and g). Finally, Δ6/FL MR ratio significantly decreased by hypotonicity (Fig. 5h) in KC3AC1 cells stably transfected with scr_shRNA (as expected, see Fig. 3c, lower panel). Importantly, this effect was partially prevented in KC3AC1 cells stably transfected with HuR_shRNA, indicating that HuR plays a crucial role in the control of Δ6 MR expression in renal cells.

## Discussion

In addition to its fundamental role in mRNA stability, HuR is also involved in RNA processing^8, 29^. For instance, HuR was shown to promote skipping of exon 6 in the transcript of the apoptosis receptor Fas, resulting in the generation of a soluble isoform that prevents programmed cell death^30^. HuR binding to the alternative 3′-terminal exon in pre-messenger target transcripts has been also shown to promote their splicing^31^. Since we have recently demonstrated that HuR constitutes a major mediator of hypotonicity-induced increase in MR expression under hypotoncity (see Lema *et al*., 2017, submitted), we speculated that HuR might also participate to the processing of MR transcript in renal cells. Using an exon-specific PCR strategy, we identified two novel MR splice variants in the renal KC3AC1 cells, notably the murine the Δ6 MR (present paper) but also another minor splice variant Δ5.6 MR. This latter Δ5.6 MR splice variant lacks exon 5 and exon 6, generating a truncated receptor lacking the hinge and LBD domains of the mouse MR (data not shown), similarly to the human Δ5.6 MR we previously discovered in the human heart^21^. Further functional characterization of the murine Δ5.6 MR indicated that it behaves as a transcription factor insensitive to aldosterone stimulation, consistent with a ligand-independent transactivating function of this LBD-lacking receptor (data not shown). Moreover, the murine Δ5.6 MR had no effect on the transcriptional activity of murine FL MR (data not shown). These results contrasted with those published in *Zennaro et al*.^21^ since the human Δ5.6 MR was shown to exert a dominant-positive effect on the transcriptional activity of human FL MR. Given the low abundance of the murine Δ5.6 MR and the absence of its functional interaction with the FL MR signaling, we did not further characterize this murine splice variant.

In sharp contrast, the murine Δ6 MR splice variant resulted from a HuR-dependent exon 6 skipping event. This splice variant encoded for a truncated MR protein, lacking the LBD, which is replaced with a shorter 26 aa C-terminal tail that is however, 90% identical to the human Δ5.6 MR splice variant^21^. Moreover, this constitutively active Δ6 MR splice variant behaves as a ligand-independent transactivator and exerts dominant-negative effects on the FL MR. More generally, a large number of naturally occurring C-terminal splice variants of other nuclear receptors (e.g. GRβ, ERβ, TRα, DAX-1) have already been identified^32^. None of them bind endogenous ligands but all display dominant-negative activity, underscoring that several mechanisms may account for the negative interfering effect of Δ6 MR on MR signaling. Considering the exclusive and ligand-independent nuclear localization of Δ6 MR (see Fig. 2a), there may be competition for binding to the hormone response elements on the DNA, with Δ6 MR homodimers or heterodimeric Δ6 MR- and FL MR-containing complexes, thereby decreasing the transcriptional activity of FL MR. An alternative, non-mutually exclusive explanation is that, as coregulators form a functional link between the liganded receptor and the basal transcription machinery, transcriptional partners, including coregulators, may be involved, accounting for the negative interference of Δ6 MR with FL MR activity. We have demonstrated that Δ6 MR splice variant is expressed in all mineralocorticoid-sensitive tissues and cells. However, the abundance of Δ6 MR relative to that of FL MR is highly variable, with a four times higher Δ6/FL MR ratio in the heart, lung and BAT than in the kidney, but with a lower quantified ratio in the brain. These findings suggest that Δ6 MR may contribute to the modulation of mineralocorticoid signaling in a tissue- and cell-specific manner. For instance, Leung *et al*. demonstrated that glucocorticoid-insensitive asthma is associated with an increase in levels of the GRβ splice variant, which antagonizes GRα^33^. Extensive analyses of Δ6 MR expression and regulation in normal and pathophysiological situations should provide greater insight into the mechanisms regulating MR-mediated signaling. It would be of particular interest to study Δ6 MR expression in mineralocorticoid resistance syndrome in the context of PHA1^3, 34^ but also under physiological situations where abnormally low levels of renal MR were reported at birth, leading to partial yet physiological aldosterone resistance in human newborns^35^. Interestingly, some of the identifed MR mutations related to PHA1 were localized in splice sites; one of these were demonstrated by *Kanda et al*., who identified an exon 7 skipping event for a familial PHA1 patient^36^. Another study conducted by Sartorato *et al*., reported that some of the mutated MR in the context of PHA1 exerted a dominant-negative effect on the FL MR^37^.

Another original finding of the present study was the demontration that the murine Δ6 MR expression is regulated by osmotic stress. Indeed, the Δ6/FL MR ratio in renal cells decreased by 50% in hypotonic conditions, but tripled under hypertonic conditions, clearly demonstrating that regulation of murine Δ6 MR and FL MR expression operates both *in vitro* and *in vivo* (see Supplementary Table S1). Interestingly, the hypertonic-induced increase of Δ6 MR expression was accompanied by a strong nuclear subcellular localization of HuR protein (Supplementary Fig. S1), suggesting that nuclear HuR localization is a prerequisite to its function on pre-mRNA processing.

With respect to the biological significance of Δ6 MR, we should mention our recent work in which we have shown that hypotonicity enhances MR signaling when KC3AC1 cells are apically exposed to hypotonic medium, whereas hypertonicity conversely leads to an impairment of renal MR signaling^7^. In addition, we unambiguously demonstrated that hypotonicity induces an increase in FL MR expression and action, leading to a significant enhancement of aldosterone-stimulated MR recruitement onto *Sgk1* promoter region compared to isotonic conditions (Lema *et al*., 2017, submitted). Collectively, these data strongly suggest that the regulation of FL MR and Δ6 MR expression and their corresponding functional activities may represent an important mechanism involved in the modulation of adaptive responses of renal cells to extracellular variations of tonicity. Likewise, a recent study demonstrated the importance of alternative splicing for adaptation to extracellular osmotic variations, as shown for the bottlenose dolphin in which a novel splice variant of the aquaporin 2 water channel was identified, ubiquitous expression of which being essential for cell resistance to sea water, an hyperosmotically environnement^38^.

Finally, we used an RNA interference strategy to demonstrate the involvement of HuR in the splicing of MR transcript. The hypotonicity-induced decrease in relative Δ6 MR levels was restrained after reduction of nuclear HuR, providing additional support for the crucial role played by HuR in controlling renal mineralocorticoid signaling. Moreover, compelling evidence showed that deregulation of HuR expression or subcellular localization leads to the developpement of renal diseases such as diabetic nephropathy, inflammation and fibrosis, highlithing its crurial role in renal pathophysiology^6^. However, we could not exclude the involvement of other splicing factors such as TIA-1/TIAR (T-cell intracellular antigen) or hnRNP C (Heterogeneous Nuclear Ribonucleoprotein C), which were described as partner or competitor in HuR-mediated exon-skipping event as previously demonstrated for Fas transcripts^39^. It remains to investigate whether these splicing factors are involved in MR editing in order to get the whole picture of posttranscriptionnal events impacting renal MR expression and activity.

**Figure 6:**
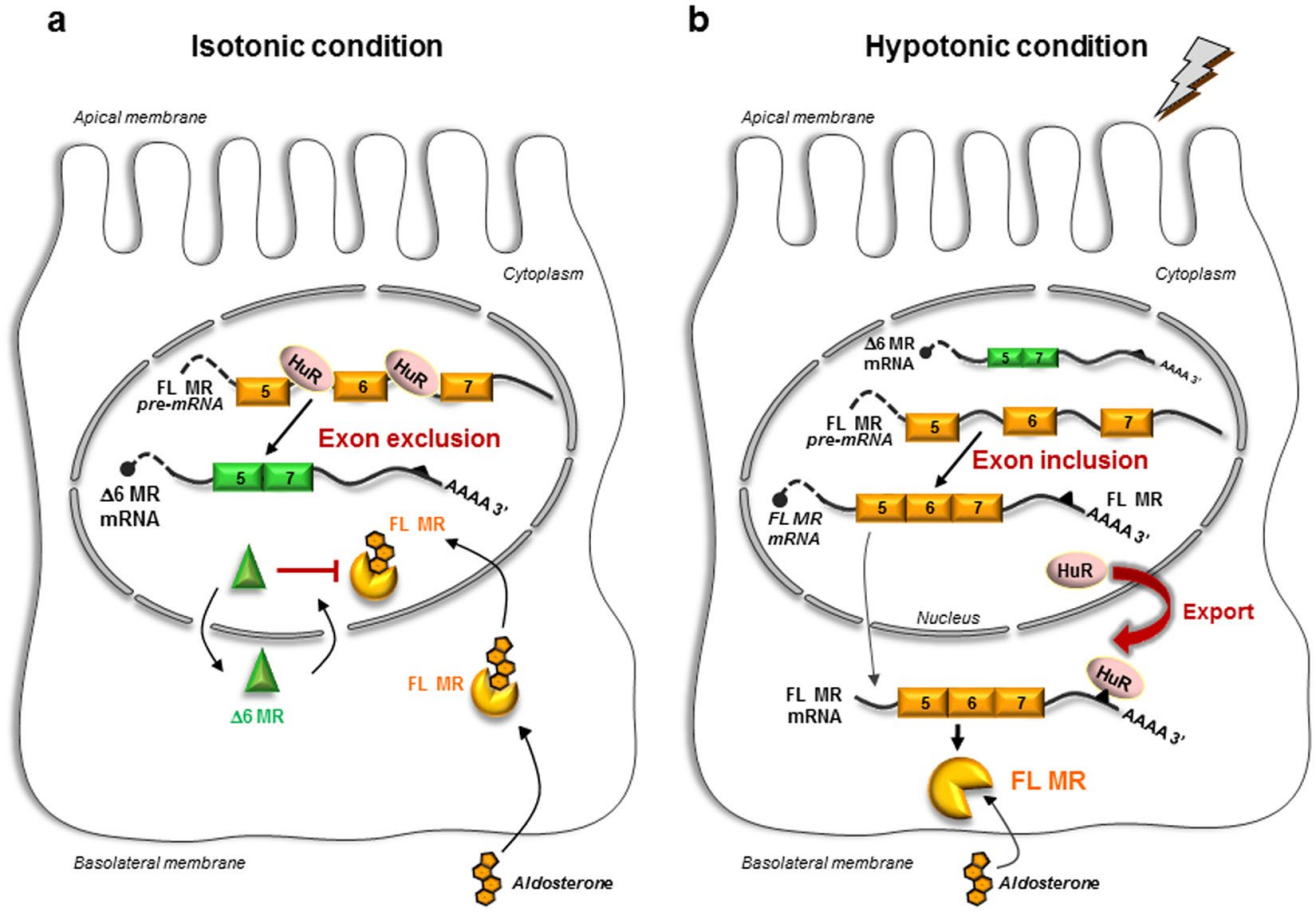
Proposed model for the posttranscriptional control of renal MR expression by HuR. (**a**) Under isotonic conditions, HuR is located in the nucleus, where it splices the MR transcript (exon 6 exclusion) to generate the Δ6 MR splice variant, which entirely lacks exon 6 of the MR transcript. The Δ6 MR protein, whose expression is regulated by tonicity, exerts dominant negative effects on the transcriptional activity of the full-length MR. (**b**) Under hypotonic conditions, HuR is rapidly exported to the cytoplasm, reducing the amount of HuR in the nucleus and facilitating generation of the full-length MR transcript (exon inclusion). In the cytoplasm, HuR stablizes the MR transcript by interacting with its 3′-UTR, thereby ultimately enhancing renal MR signaling. FL: full-length; MR: mineralocorticoid receptor; HuR: Human antigen R.

In conclusion, our findings shed new light on the fascinating dual role of HuR as a master regulator of MR expression under osmotic stress, through both MR transcript stabilization and alternative splicing control. These mechanisms converge to potentiate aldosterone responses in renal cells depending on osmotic stress challenge. We propose a model in which HuR is mostly present in the nucleus under isotonic conditions (Fig. 6) and behaves as a splicing modulator, promoting the removal of exon 6 from the MR pre-mRNA. This alternative splicing gives rise to the Δ6 MR splice variant, a ligand-independent transactivator exerting dominant-negative effects on FL MR-mediated transcriptional activity. Conversely, hypotonicity triggers the rapid, transient and reversible export of HuR to the cytoplasm, decreasing the amount of nuclear HuR, (see Lema *et al*., 2017, submitted) promoting exon 6 inclusion and favoring the FL MR mRNA synthesis. Concomitantly, cytoplasmic HuR binds MR 3′-UTR, notably on a specific hairpin secondary structure, thus enhancing MR expression and signaling and potentiating renal aldosterone responsiveness (see Lema *et al*., 2017, submitted). We therefore suggest that HuR is a physiological regulator of renal MR abundance, which is a key molecular determinant for the MR-mediated signaling pathway. We also describe an osmoregulatory loop in which expression and function of a steroid receptor that controls sodium homeostasis is posttranscriptionally regulated by variations of tonicity and by RBP action. Because MR expression is profoundly altered in various pathophysiological states^4, 40^, it will be important to determine whether such mechanisms occur in other nephronic segments and in other MR-expressing cells (colon, inner ear, heart, neurons, etc.). In this context, other stresses (oxygen depletion, oxidative stress, energy shortage) which are known to modify HuR subcellular localization^8^ and Tis11b expression^41^ might probably be involved in the rapid regulation of MR expression and splicing, and, ultimately, in the control of mineralocorticoid-related signaling pathway.

## Methods

### Cell culture

HEK293T and COS7 cells were cultured in DMEM medium supplemented with 2 mM glutamine, 20 mM HEPES, pH 7.4, 100 U/ml penicillin, 100 µg/ml streptomycin, and 10% fetal calf serum (Biowest). All reagents were from Life Technologies unless otherwise stated. KC3AC1 cells were cultured under isotonic conditions, at 300 mOsmol/l, as previously described^42^. Hypotonic conditions (150 mOsmol/l) were achieved by two-fold dilution of the medium in sterile water. Hypertonic conditions (500 mOsmol/l) were achieved by adding Raffinose (Sigma Aldrich) to the medium at 0.2 M final concentration. Minimal medium lacking dexamethasone (Sigma Aldrich), epidermal growth factor (EGF, Peprotech), triiodothyronine (T3, Sigma Aldrich) and dextran charcoal-coated (DCC) serum was used to study aldosterone action (Sigma Aldrich).

### Plasmid constructs

The FL coding sequence of the mouse MR (*mMR*, *Nr3c2*) was obtained by amplifying the renal cDNA by PCR and inserting it into the pcDNA3.1 vector (Thermo Fisher). Long site-directed mutagenesis of mouse Δ6 MR was performed with the Q5 Site-Directed Mutagenesis kit (New England Biolabs), with specific primers (Supplementary Table S2) designed with the NEBaseChanger website (http://nebasechanger.neb.com/), using the FL mMR-pcDNA3.1 plasmid as the template.

### Transfection assays

COS7 cells were cultured for 24 hours in the presence of complete medium supplemented with 10% DCC. Transfections for immunocytochemistry were performed in 4-well chamber slides (Sarstedt), with 500 ng of FL MR- or Δ6 MR-encoding vector, in the presence of Lipofectamine 2000. The functionality of mouse FL MR and Δ6 MR were assessed by transfecting HEK293T cells with FL MR (20 ng) and Δ6 MR (0, 20 or 40 ng) expression vectors and plasmids coding for β-galactosidase (pMiR-βgal, 35 ng) or Firefly-luciferase (pF31-Luc, 40 ng) and pcDNA3 plasmid (0, 20 or 40 ng) which were used to transfect the same amount of total plasmid DNA per condition. Then the transfection medium was replaced, 6 hours after transfection, with medium supplemented with 10% DCC. The following day, cells were cultured for 24 hours in the absence or presence of aldosterone (0.1 nM or 10 nM). Forty eight hours post-transfection, cells were lysed with Passive 1X Lysis Buffer (Promega) for 30 minutes with shaking, and firefly-luciferase activities were determined and normalized relative to β-galactosidase activities.

### Quantitative RT-PCR

Total RNA were extracted from cells or tissues with TRI Reagent (Euromedex) and a TissueLyser (Qiagen) was used for organs. Tissues samples were collected from wild-type mice which were bred according to the Guide for the care and use of Laboratory animals as described in the Study Aproval section. Mice were euthanized at the age of eight-week old before the collection of different organs: kidney, heart, lung, brain and brown adipose tissue. Cells extracted were provided from different cell lines established in the laboratory, the hibernoma-derived T37i cells, the cortical collecting duct KC3AC1 cells and the ES-derivated neurons, already described in previous studies^42–44^. One µg of total RNA samples were first treated with DNase I (New England Biolabs) and then were reverse-transcribed into cDNA with the High Capacity cDNA reverse transcription kit RT-qPCR. Quantitative PCR were performed with specific primers and Power SYBR® Green PCR Master Mix (Life Technologies), a QuantStudio^TM^ 6 Flex Real-Time PCR System was used to quantify transcript expression. Relative expression in a given sample was calculated as amol of the specific gene/fmol of *18S*.

### Taqman assays

cDNA samples obtained from cells and tissues RNA extracts were described above. These samples were analyzed with Taqman® Universal Master Mix Reagent (Life Technologies), with specific primers and probes (see Supplementary Table S2) and the QuantStudio^TM^ 6 Flex Real-Time PCR System. Relative expression was calculated for each sample as the amol of the gene of interest/fmol of *18S* or amol of *Gapdh*. The specificity of each primer/probe set was confirmed by preliminary experiments with plasmid constructs corresponding to FL MR or Δ6 MR. No cross-reaction was observed between the FL MR primers and Δ6 MR probe or between the Δ6 MR primers and FL MR probe, confirming that each primer/probe set amplified only its specific target.

### RNA interference

We generated KC3AC1 cell lines stably expressing shRNA, by transfecting cells with 10 µg of HuR shRNA-encoding plasmid (#TRCN0000112085 or #TRCN0000112087, Sigma Aldrich) or a control (scrambled) shRNA-encoding plasmid (#SHC002), in the presence of Lipofectamine 2000. Then, we added 1 µg/ml puromycin, to the medium for the selection of transfected clones 24 hours after transfection. Clonal cells were obtained by limiting dilution of the cell suspension and were routinely cultured in the presence of puromycin.

### *In vitro* translation analysis

FL MR or Δ6 MR plasmids (1 µg) was translated *in vitro* with the TnT-T7 Quick Coupled Transcription/Translation kit (Promega) and the products of the reaction were analyzed by western blotting.

### Western blot analysis

Proteins from reticulocyte lysates or differentiated KC3AC1 cells were extracted with a lysis buffer (50 mM Tris HCl, pH 7.5, 150 mM NaCl, 5 mM EDTA, 30 mM sodium pyrophosphate, 50 mM sodium fluoride, 1% Triton X-100, 1% protease inhibitors) and the debris were removed by centrifugation. Protein samples were subjected to SDS-PAGE and processed for the multiplex detection of HuR or MR protein, together with α-tubulin or β-actin protein as a loading control. Signal fluorescence intensity was determined with an Odyssey® Fc (Li-Cor). Detailed information about the antibodies used is provided in Supplementary Table S3.

### Immunocytochemistry

KC3AC1 and COS7 cells were cultivated and transfected in 4-well chamber slide (Sarstedt). Cells were fixed with 4% paraformaldehyde (Electron Microscopy Sciences). Immunocytochemistry was performed as described in ref. 26 with the antibodies mentionned in Supplementary Table S3. Immunodetection of MR and HuR protein were performed with Alexa 555- coupled secondary antibody and nuclear counterstaining was performed with 0.5 μg/ml DAPI. Cells were observed with the Olympus BX61 and images were acquired at 40X using a Retiga-2000R.

A specific quantification of cytoplasmic and nuclear intensities of HuR were performed using an automated high-throughput microscopy (HTM), precision in the calculation method is described in ref. 45. The bioapplication Target Activation Bioapplication was used to quantify HuR protein levels in KC3AC1 cells expressing Scr_ or HuR_shRNA. Briefly, a secondary nuclear mask was created based on the the primary nuclear mask (DAPI) and the mean fluorescence intensity within this mask was determined from mean values obtained for >5000 cells.

### Homology modelling

The sequence of the mouse FL and Δ6 MR LBDs were aligned to that of the human MR. Theses sequence alignments were then used together with the X-ray crystal structure of the human MR LBD (Protein Data Bank identification number 2AA2)^25^ as templates to generate the three-dimensional homology models of the FL and and Δ6 MR using the Modeller9v8 program^46^.

### Investigations in mice

Animals were provided with free access to water, except for the water deprivation group, which did not receive any water for 18 hours. Mice were subjected to either a high Na^+^ diet (Genestil) for two weeks or they received an i.p. injection of furosemide (40 mg/kg of body weight, Renaudin) over a period of 3 hours. For water intoxication, animals were fed orally for 6 hours with 3% vol/kg of BW of a 10 mM glucose solution prepared with tap water (approximately 800 μl/mouse), *via* gavage feeding needles (Phymep). Animals (*n* = 6–7 per group) were euthanized and kidneys were collected and snap-frozen in liquid nitrogen for subsequent analyses.

### Study approval

Mice were bred in accordance with the Guide for the Care and Use of Laboratory Animals published by the National Institutes of Health (NIH Publication No. 85-23, revised 1996). The animal facility was approved (no. C94-043-12) by the French Ministry of Agriculture. All procedures were approved by the local ethics committee, CAP Sud (No. 2012-021).

### Statistics

Data represent means ± SEM. One-way ANOVA or Mann Whitney U-tests were used, as appropriate, to assess the significance of differences (Graphpad Prism software). A *P*-value of 0.05 was considered statistically significant: *P < 0.05; **P < 0.01; ***P < 0.001.

## Electronic supplementary material

Supplementary figure and tables
