# Supplementary material for: HuR-Dependent Editing of a New Mineralocorticoid Receptor Splice Variant Reveals an Osmoregulatory Loop for Sodium Homeostasis
Source: Sci Rep. 2017 Jul 6;7:4835. doi: 10.1038/s41598-017-04838-8 (PMC5500589; doi:10.1038/s41598-017-04838-8)
Supplement: Supplementary file 1 — Supplementary figure and tables [file 41598_2017_4838_MOESM1_ESM.pdf]

HuR-Dependent Editing of a New Mineralocorticoid Receptor Splice Variant Reveals an Osmoregulatory Loop for Sodium Homeostasis

Ingrid Lema[1], Larbi Amazit[1,2], Khadija Lamribet[1], Jérôme Fagart[1], Anne Blanchard[3], Marc Lombès[1,4¶], Nadia Cherradi[5,6,7¶], Say Viengchareun[1¶].

¶ Senior authors who equally contributed to this work

[1] Inserm U1185, Fac Med Paris Sud, Université Paris-Saclay, F-94276, Le Kremlin-Bicêtre, France;
[2] UMS 32, Le Kremlin Bicêtre, 94276, France;
[3] Inserm, Centre d'Investigations Cliniques 9201, F-75015 Paris, France;
[4] Assistance Publique-Hôpitaux de Paris, Hôpital de Bicêtre, Service d'Endocrinologie et des Maladies de la Reproduction, Le Kremlin Bicêtre F-94275, France;
[5] Institut National de la Santé et de la Recherche Médicale, Inserm U1036, Grenoble, France, F-38000
[6] Commissariat à l'Energie Atomique et aux Energies Alternatives, Institut de Biosciences et Biotechnologies de Grenoble, Laboratoire Biologie du Cancer et de l'Infection, Grenoble, F- 38000, France;
[7] Université Grenoble Alpes, Unité Mixte de Recherche-S1036, Grenoble, F-38000, France.

Correspondence should be addressed to:

Marc Lombès, MD, PhD or Say Viengchareun, PhD
Inserm U1185, Faculté de Médecine Paris-Sud
63 rue Gabriel Peri, F-94276, Le Kremlin-Bicêtre, France
E-mail: marc.lombes@u-psud.fr or say.viengchareun@u-psud.fr
Phone: 33 1 49 59 67 02
Fax: 33 1 49 59 67 32

Or

Nadia Cherradi, PhD
INSERM U1036, BCI
Institut de Biosciences et Biotechnologies de Grenoble, CEA Grenoble
17, rue des Martyrs
38054 Grenoble Cedex 09, France
E-mail: nadia.cherradi@cea.fr
Phone: 33 4 38 78 35 01
Fax: 33 4 38 78 50 58

Running headline: Tonicity dictates HuR-dependent MR transcript editing

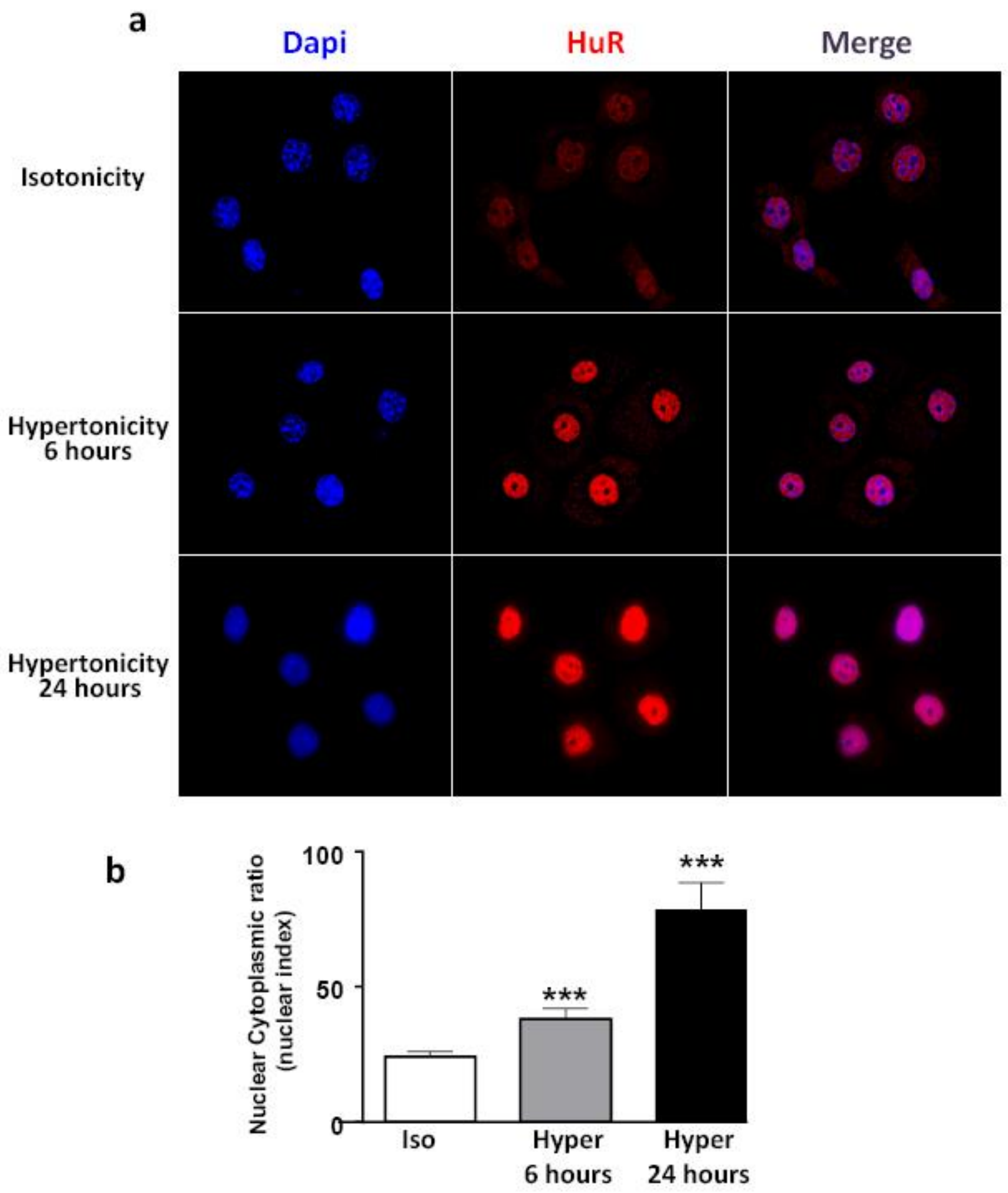

Figure S1. Hypertoncity increases HuR nuclear localization in renal cells.

KC3AC1 cells were incubated for 6 or 24 hours under isotonic (Iso) or hypertonic (Hyper) conditions. Immunocytochemistry for HuR was performed as described in the Methods section, with the 3A2 anti-HuR antibody. The nuclei were stained with DAPI (blue). Representative images of the subcellular localization of HuR (Supplementary Fig. S1a). HuR nuclear staining was increased under hypertonic conditions compared to isotonic conditions. This result was corroborated by the subcellular trafficking of HuR protein quantification with a high-throughput microscopy (HTM) analysis, as shown in Supplementary Fig. S1b. The results are expressed as the mean ratio of nuclear to cytoplasmic fluorescence ± SEM (*n*>2800 cells). Bar, 25 µm. ****P*<0.001; NS=not significant (Mann-Whitney U-tests).

Supplementary Table S1

Summary of the functional consequences in terms of FL MR and Δ6 MR expression in response to osmotic stresses *in vitro (*KC3AC1 cells) and *in vivo (*Challenges in mouse models*)*.

Stress	Luminal osmolarity	Na delivery	FL MR	Δ6 MR	Δ6/FL ratio
**KC3AC1 cells**					
Hypertonicity	↑	-	→	↑	↑↑
Hypotonicity	↓	-	↑	→	↓
In vivo					
High Na^+ intake	↑	↑	→	↑	↑
Furosemide	↑↑	↑	↓	↑↑	↑↑
Water deprivation	↑	→	→	→	→
Water load	↓	→	→	↓	↓

Supplementary Table S2

All primer sequences are shown 5' to 3'.

Primer sequences used for site-directed mutagenesis to generate the mutR3 mutant

Name	*Forward primer*	*Reverse primer*
Δ6 MR	AGAGAAGATGCATCAGTCTG	CTGGAAGTACCTTGGCCC

Primer sequences used in RT-qPCR

Name	*Accesion number*	*Amplicon size (bp)*	*Forward primer*	*Reverse primer*
HuR (*Elavl1*)	NM_010485.3	87	CAGCCAATCCCAACCAGAA	TGGTGTACAGGGCCTCCAAA
36b4 (*Rplp0*)	NM_007475.5	128	AGCGCGTCCTGGCATTGTCTGT	GGGCAGCAGTGGTGGCAGCAGG

Oligonucleotide sequences of forward (f) and reverse (r) primers and Taqman probes (p)

cDNA	*Accession number*	*Primer*	*Sequence (5'-3')*
FL MR	NM_001083906.1	f	TCGTTTGCCTTGAGTTGGAGAT
		r	TGCATCAGTCTGCCATGTATGA
		p	TACAAACATACGAACAGCC
Δ6 MR	NM_001083906.1	f	CAGATGATCCAAGTCGTGAAGTG
		r	GTATGAGCTGTGCCAGGGGA
		p	TACTTCCAGAGAGAAGATG
18s	NR_003278.3	f	TAGAGGGACAAGTGGCGTTCA
		r	TGTGATGCCCTTAGATGTCCG
		p	ACCCGAGATTGAGCAAT
Gapdh	NM_008084.3	f	Unknown (provided by Thermofisher Scientific)
		r /p	Mm99999915_g1

Supplementary Table S3

Antibodies for western blot analysis

Name (Provider)	*Species*	*Protein*	*Molecular weight (kDa)*	*Dilution*
Primary Antibodies				
39N	Rabbit	MR	130 kDa	1:1000 or 1:5000 (tissues)
α-tubulin (Sigma)	Mouse	α-tubulin	50 kDa	1:10000
3A2 (Santa Cruz)	Mouse	HuR	36 kDa	1:500
Secondary Antibodies				
Dylight Anti-Rabbit 800 (Fisher Scientific)	Goat	Rabbit IgG		1:15000
Dylight Anti-Mouse 680 (Fisher Scientific)	Rabbit	Mouse IgG		1:15000
Dylight Anti-Rabbit 680 (Fisher Scientific)	Goat	Rabbit IgG		1:15000
Dylight Anti-Mouse 800 (Fisher Scientific)	Rabbit	Mouse IgG		1:15000

NB: TBST (50 mM Tris-HCl pH 7.4, 150 mm NaCl, 0.1% Tween 20), Blocking Buffer (Li-Cor)

Antibodies for immunohistochemistry

**Name (Provider)**	**Species**	**Protein**	**Dilution**
Primary Antibodies			
3A2 (Santa Cruz)	Mouse	HuR	1: 500
39N	Rabbit	MR	1: 736
Secondary Antibodies			
Alexa 555 Anti-Mouse (Life Technologies)	Goat	Mouse IgG	1:15000
